# Surface Evaluation of a Multi-Pass Flexible Magnetic Burnishing Brush for Rough and Soft Ground 60/40 Brass

**DOI:** 10.3390/ma13194465

**Published:** 2020-10-08

**Authors:** Ayman M. Alaskari, Abdulaziz I. Albannai, Meshal Y. Alawadhi, Abdulkareem S. Aloraier, Tatiana Liptakova, Abdullah A. Alazemi

**Affiliations:** 1 Department of Manufacturing Engineering Technology, College of Technological Studies, PAAET, Shuwaikh 70654, Kuwait; ai.albannai@paaet.edu.kw (A.I.A.); my.alawadhi@paaet.edu.kw (M.Y.A.); as.aloraier@paaet.edu.kw (A.S.A.); 2 Department of Material Engineering, Faculty of Mechanical Engineering, University of Zilina, 10 26 Zilina, Slovakia; tatiana.liptakova@fstroj.uniza.sk; 3 Mechanical Engineering Department, College of Engineering and Petroleum, Kuwait University, P.O. Box 5969, Safat 13060, Kuwait; a.alazemi@ku.edu.kw

**Keywords:** multi-pass, flexible magnetic burnishing brush, surface integrity, brass, permanent magnets

## Abstract

Burnishing is an advanced finishing process that produces higher-quality surfaces with better hardness and roughness than conventional finishing processes. Herein, a flexible magnetic burnishing brush comprising stainless steel pins under permanent magnet poles was used to investigate the influence of multiple passes and directions on the produced surface of soft and rough ground prepared brass. In total, five different samples were burnished on each of the two brass samples prepared. Four samples were processed in the same direction for up to four passes and the fifth sample was processed with two passes in the opposite direction. Results indicate that there was approximately a 30% increase in hardness and an 83% increase in microroughness for rougher-surface brass samples. For smoothly prepared surfaces, there was approximately a 14% increase in hardness and a 35% increase in microroughness. In the same direction of multi-pass burnishing, increasing the number of passes negatively affected surface roughness; for rougher surfaces, the surface hardness reduced and process uniformity increased owing to surface over-hardening and flaking mechanisms, and for smoother surfaces, the hardness, roughness, and process non-uniformity increased with the number of passes owing to repeated surface deformation at some locations and high flaking at other locations. Compared to single-pass burnishing, wherein the surface roughness and microhardness showed almost no change with high process uniformity, in burnishing with two opposite-direction passes, the produced surface exhibited better surface roughness, process uniformity, and microhardness improvements owing to a reverse strain mechanism. Hence, opposite burnishing passes are recommended.

## 1. Introduction

All machined surfaces have inherent peaks and valleys, known as surface roughness, which are produced by tools and other factors such as vibrations in machine structures, wear, and process parameters. The surface roughness of any machined part is a measurable quantity that directly influences the geometry of the machined surface along with the overall texture of the final surface produced, including the micro-irregularities on the surface [1]. In addition, surface roughness affects a few important mechanical properties of machined parts such as fatigue behavior, corrosion resistance, microhardness, and creep life [2]. Thus, some conventional machining processes that depend on chip removal, such as turning and grinding can produce surfaces with high surface roughness; such surfaces are of low quality owing to surface abrasion and geometric tolerance problems caused by machined chips [3]. Quality and productivity are the main challenges of metal-based industries that aim to produce parts with high geometrical accuracy and better surface finishes, ensuring reliable performance of the parts produced. Therefore, some conventional machining processes cannot satisfy the industrial scope, and a post-machining surface finishing process is required to achieve a high-quality surface finish [4]. Post-machining surface finishing methods, such as burnishing, which is based on the cold working principle, are employed to achieve good surface roughness along with other added advantages such as high microhardness, wear resistance, fatigue strength, and corrosion resistance [5]. Burnishing is a mechanical surface modification technique and chipless operation that can be utilized in various industries as a final finishing process to generate smooth high-quality surfaces or surfaces with specific structures [3,6]. The process exerts severe plastic deformation on surface irregularities to decrease the distance between the peaks and valleys, which helps enhance surface quality. Furthermore, the surface of the material is work-hardened, and the microstructure of the work surface is refined [7]. Burnishing is intentionally employed to induce compressive residual stresses on the surface of load-bearing engineering components such as the landing gear, leaf springs, pipeline seams, and turbine blades [8]. Maximum residual stress is observed when the burnishing pressure force reaches its maximum value and other input parameters reach their minimum [9]. Teimouri and Amini [10] stated that burnishing could also be used to remove micro-cracks and voids remaining from previous production processes. Burnishing can improve the fatigue life and mechanical properties of parts by inducing compressive residual stress on the work surface [11]. A few researchers [9,12] have indicated that the results obtained from the analysis of variance demonstrated that the burnishing force has a significant influence on both surface hardness and roughness. Surface integrity is crucial in the machining process because it is used to evaluate the high stress and the loaded components on the machined surface [13]. Dzierwa and Markopoulos [9], for instance, investigated the effect of the ball burnishing process on the surface topography of 42CrMo4 steel samples and demonstrated that the root mean square height of the surface (Sq) could be reduced from 0.522 to 0.051 μm and wear resistance could be increased to be higher than that of ground samples. Moreover, burnishing can be used on different types of materials such as steels (alloy steel), magnesium alloys, aluminum alloys, titanium alloys, cobalt-chromium alloys, and brass [6]. Another study of surface hardness involving burnishing parameters revealed that the normal force and number of passes are the most important factors for reducing surface roughness [14]. Therefore, burnishing has been successfully used and adopted widely as a post-machining process to achieve a high-quality surface finish. However, despite its great potential and advantageous features, burnishing exhibits several limitations related to the surface and dimensional accuracies. For instance, if the finishing material is a wire or has a micro-scale diameter, a high pressure is required to implement burnishing. However, such high pressures could damage the surface that requires finishing, and hence burnishing cannot be implemented [15].

Magnetic field-assisted manufacturing methods are comparatively new finishing methods that are effective for cleaning, finishing, burnishing, and deburring metals or even advanced engineering material parts [16]. These processes are effective, and their implementation is highly flexible for the economical machining or surface finishing of components. Moreover, they can be applied irrespective of part hardness, providing high surface quality and precision without imparting additional residual stresses to the finished surface [17]. Thus, in magnetic-aided burnishing (MAB), a burnishing force is induced when a strong magnetic field and relative speed are realized between the magnetic burnishing particles (MBPs) and the material surface. There are four primary MAB techniques: roller burnishing, ball burnishing, diamond burnishing, and burnishing using a flexible magnetic burnishing brush (FMBB). Hassan [18] presented roller and ball burnishing, where he demonstrated that tools, the burnishing force, and the number of burnishing tool passes can be optimized to improve surface roughness and increase the surface hardness of commercial alloys and brass. Okada et al. [19] compared the proposed diamond burnishing method with conventional methods. They proved that their proposed method helps obtain high-quality burnished surfaces with smooth profiles, low surface roughness, and high glossiness for stainless steel workpieces. Aalaskari et al. [20] proved that their FMBB can be applied to cylindrical magnetic stainless steel pins under only permanent magnetic poles to improve the surface quality of 60/40 brass. Burnished brass exhibited better surface hardness as well as roughness compared with unburnished brass, while the corrosion resistance improved only for selected relative burnishing speeds owing to surface uniformity. In addition, some attractive advantages of the burnishing process include decreasing roughness, increasing hardness, generating compressive stresses that contribute to improved mechanical properties such as strength, and elimination of chips that can be produced with other traditional processes, making it a green process [21,22]. In addition to improving fatigue life, wear and corrosion resistances, surface quality, and mechanical properties, magnetic burnishing provides an economic and environmental surface finishing process, which attracts many industries searching for high-quality product performance and life.

The present study is a continuation of the FMBB technique which was the novelty of Aalaskari et al.’s [20] work, and mainly was deployed by using two permanent magnets to form a magnetic field to control the stainless steel particles to roll over the required ground surface. Thus, the aim of current study is to examine the influences of multi-passes and directions of FMBB on both soft and rough ground brass surfaces. Most of the important surface properties such as roughness, microhardness, and uniformity are being investigated to build up the best beneficial situation from the number of passes and directions when the FMBB is operated.

## 2. Experimental Section

### 2.1. Material Preparation and Setup

Ten 60/40 yellow brass (C274) plates with dimensions of 90 × 90 × 3 mm were used as workpieces. Five of those plates were prepared by grinding them on a belt surface grinder (Rajendra Metal Industriesr, Mumbai, India) consisting of 180-grit aluminum oxide and the other five workpieces were prepared by grinding them on a belt surface grinder (Buehler, Pliensauvorstadt, Germany) consisting of 400-grit aluminum oxide. Workpieces were thoroughly cleaned with desalted water and then with acetone and allowed to dry. Cold-rolled austenitic stainless steel (grade 303) particles (pins) 0.3 mm in diameter and 5 mm in length were used as MBPs for the multi-pass burnishing process. The chemical compositions of C274 yellow brass workpieces and 303 stainless steel pins are listed in Table 1.

### 2.2. Multi-Pass Burnishing Processes

A universal milling machine (Emtex Machinery, New Delhi, India) equipped with a frequency inventor was used in all burnishing processes in this work. The frequency inventor was used to reduce the minimum allowable feed rate of the saddle (table) of the machine. With this inventor, a 2.8 mm/min minimum feed rate could be achieved.

A cylindrical permanent magnet (rare earth neodymium magnet, N52, Henzhen Zhenxing Communication Shares, Guangdong, China) with a diameter of 25 mm was placed inside the collecting chuck of the milling machine where the north pole faced the milling table; hence, the magnet could rotate with the revolutions of the spindle. N52 is the strongest permanent magnet available commercially and comprises iron, neodymium, and boron. The south N52 magnet pole, with dimensions of 90 × 90 × 10 mm, was fixed in a vice on the machine table to be located below the C274 brass workpiece.

**Figure 1:**
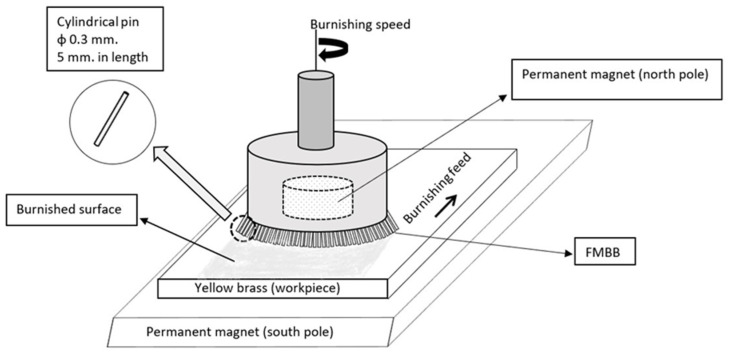
Schematic set-up for the multi-pass burnishing process [20].

A brass workpiece that was prepared by grinding with 180-grit aluminum oxide was placed above the south pole magnet and fixed to the vice. Stainless steel (303) pins were used as MBPs filled in a 4 mm gap between the north pole of the magnet and the workpiece ground surface. A speed of 1000 rpm and a feed rate of 12 mm/min with no lubricant were set as the working conditions, as shown in Figure 1. The speed and feed rate were optimized from the previous work of Alaskari et al. [20] and maintained as constant for all the burnishing processes implemented in the current study. The burnishing process, using an FMBB, was performed using a single pass, as proposed by Alaskari et al. [20]. In the current work, the workpiece with the initial preparation (180 grit) was subsequently burnished using one, two, three, and four consecutive burnishing passes. All multiple passes were processed in the same burnishing direction. A fifth workpiece was burnished using two consecutive opposite-direction passes. The other five workpieces, which were ground with 400 grit, were treated with the same burnishing process as for the previous five workpieces. The entire multi-pass process is summarized in Table 2.

### 2.3. Experimental Tests

**Figure 2:**
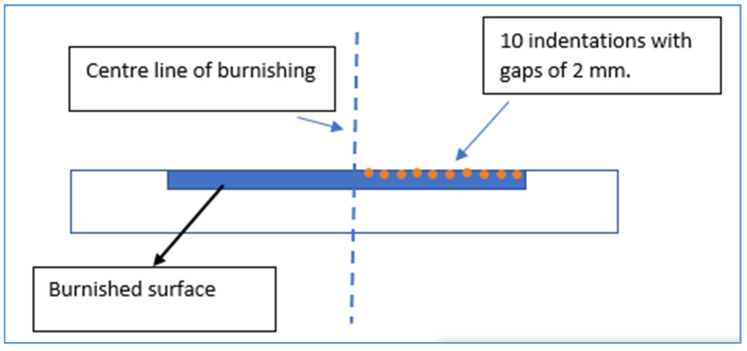
Cross-sectional view indicating the locations of indentations taken for microhardness evaluation.

An Innovatest 400 series testing machine (INNOVATEST, Maastricht, The Netherlands) was used to evaluate the Vickers microhardness (HV) of the brass workpiece surface with a force of 0.1 kgf. Ten indentations with a gap of 2 mm were taken starting from the burnishing center, forming a line toward the unburnished surface, as shown in Figure 2. This was done following the ASTM E384 standard [23] for all workpieces.

For the single pass and four passes, the top surfaces of the workpieces ground with 180 grit were evaluated and compared with the unburnished surfaces using an optical microscope (ZEISS. Oberkochen, Germany), after being thoroughly cleaned with desalted water and then with acetone and allowed to dry. This was repeated for the same conditions but for the 400-grit preparation. These evaluations took place in two locations: at 5 and 15 mm away from the center line of the burnished surface, where the burnishing surface width was approximately 42 mm.

Moreover, the surface microroughness for all workpieces, including only ground conditions, was evaluated for the ground surface using atomic force microscopy (Agilent, Santa Clara, USA) at different locations. An Agilent 5500 AFM was used to acquire 2D and 3D surface images and measure the arithmetic mean (S_a_) and root mean square (S_q_) surface roughness of the samples. AFM scanning via the contact mode and tapping mode was performed at room temperature using a rectangular AFM cantilever probe (NanoSensors, with a tip radius less than 10 nm, length of 225 μm, width of 28 μm, and thickness of 3 μm). AFM measurements were repeated multiple times on different areas of the sample, and comparable results were obtained and reproduced.

## 3. Results

### 3.1. Microhardness

**Figure 3:**
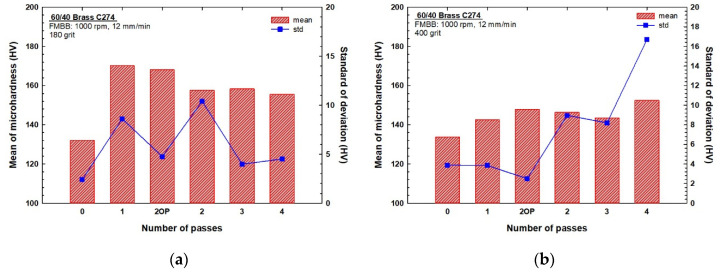
Microhardness values (means and standard deviations) for different burnishing passes ground with (**a**) 180 grit and (**b**) 400 grit.

The obtained results for microhardness are shown in Figure 3 for both initial surface preparations of (a) 180 and (b) 400 grit, where “0 pass” stands for an unburnished sample and “2OP” stands for two opposite-direction passes. Hardness evidently increased in all workpieces owing to the burnishing process under both conditions. Further, Figure 3a shows that workpieces prepared with a grit of 180 and then processed for one pass and two opposite passes improved the microhardness by 30.9% and 29.3%, respectively, compared with that exhibited by the unburnished workpiece. These two cases have optimum hardness values among the samples. Two, three, and four passes resulted in lower improvements of 19.4%, 20.1%, and 17.8%, respectively. In general, microhardness values decreased with the number of passes with a minor exception for the third pass, where a slight increase in microhardness was observed.

The standard deviations for the microhardness of samples initially ground with 180 grit were calculated, as shown in Figure 3a. This provides a good measurement of the process uniformity of the surface along the path of the burnishing brush [20]. Figure 3a shows that the two opposite passes exert better uniformity than the single pass, whereas the two passes exhibit the lowest uniformity of hardness compared with other passes. In contrast, the highest uniformity is exhibited after three and four burnishing passes. 

Figure 3b shows the microhardness values for different burnishing cases and passes for brass samples that were initially ground with 400 grit. The hardness obtained after all passes is considerably higher than that of the unburnished workpiece. The largest hardness improvement is attained after four burnishing passes and an improvement of 14% is observed. Hardness improvements of 10.5%, 9.3%, 7.3%, and 6.5% were achieved after two opposite passes, two passes, three passes, and one pass, respectively.

The hardness of the initially ground workpieces with 400 grit increases with the number of passes but decreases after three passes; further, the hardness abruptly increases after the fourth pass. A brass powder chip on the surface of the workpiece was observed after the third and fourth passes. In addition, except for the two opposite passes, the standard deviation of the microhardness increases with the number of passes; therefore, less uniformity is obtained. The two opposite passes exhibit the highest surface hardness uniformity, whereas the three passes exhibit the lowest surface hardness uniformity.

### 3.2. Microscopic Topography

**Figure 4:**
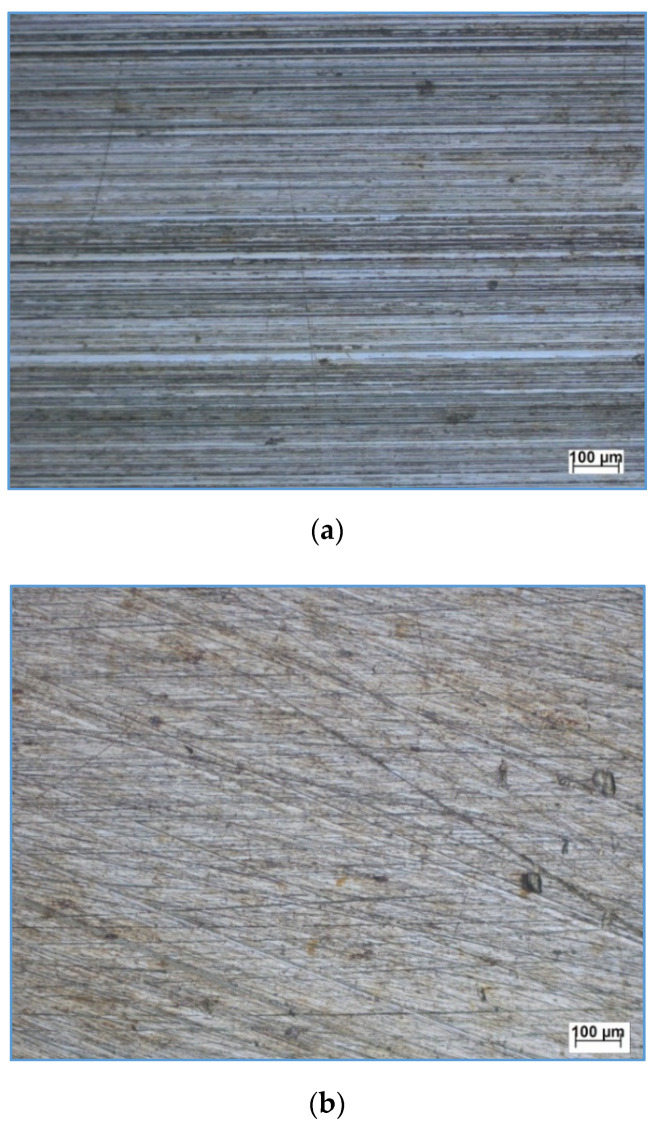
Top surface microscopy for the ground surface for (**a**) 180 grit and (**b**) 400 grit.

Figure 4 shows the top microscopy surfaces of the ground 180 grit and 400 grit, which were prepared to ensure surface uniformity before the burnishing process was implemented. Figure 4a evidently shows that the surface marks due to grinding with 180 grit are thicker and wider than those of 400 grit shown in Figure 4b.

**Figure 5:**
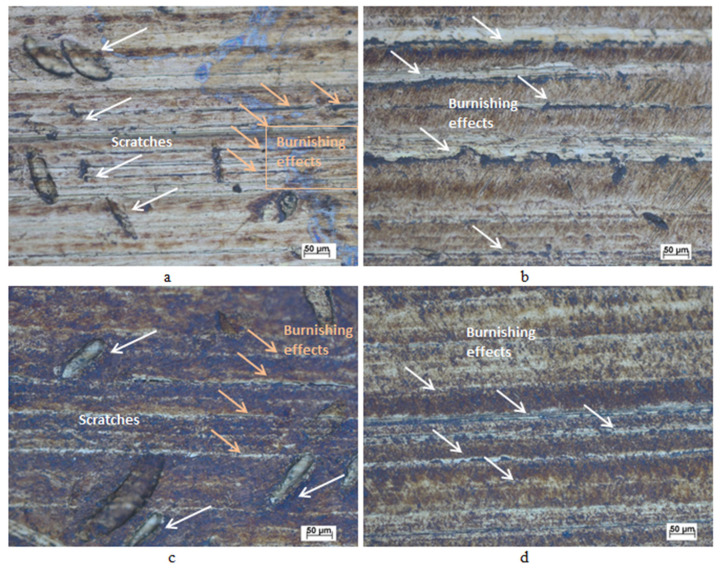
Top surface microscopy for 180-grit burnished surfaces at different passes and locations (**a**) A single pass 5 mm from the center; (**b**) a single pass 15 mm from the center; (**c**) four passes 5 mm from the center; (**d**) four passes 15 mm from the center.

Figure 5 shows the top surface microscopy for brass workpieces that were initially ground with 180 grit and then burnished for one pass (a) 5 and (b) 15 mm away from the center line of burnishing, as shown in Figure 2. The effects of the burnishing process on the peaks were lower and the number of scratches observed was higher 5 mm away from the center line of burnishing, as shown in Figure 5a, compared with those observed 15 mm away from the center line of burnishing, as shown in Figure 5b. The scratches might be due to the low initial pin velocities and non-uniformity at the location near the burnishing center before the formation of the magnetic brush. Figure 5 shows the top surface microscopy for brass workpieces initially ground with 180 grit and then burnished for four passes (c) 5 and (d) 15 mm away from the center line of burnishing. A close inspection of Figure 5c,d shows that the effects of the burnishing process on the peaks after four passes were lower than those after one pass, as shown in Figure 5a,b. The figures also evidently show that more scratches are produced near the center line of burnishing after four passes than those produced after one pass, as shown in Figure 5a,b.

**Figure 6:**
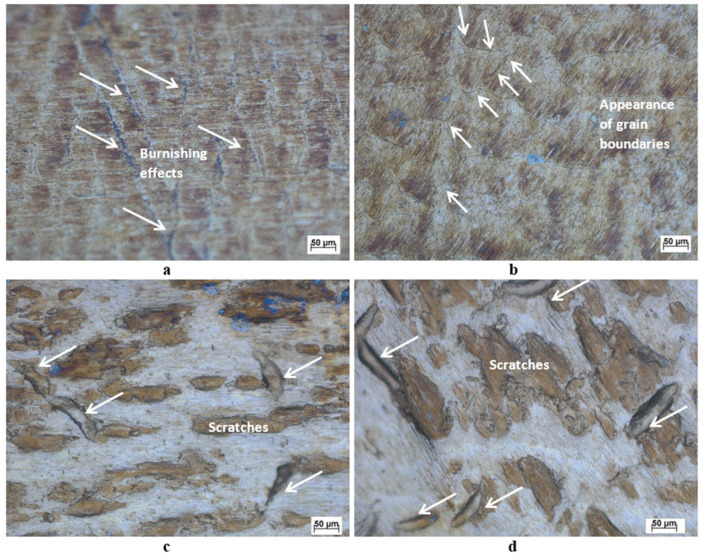
Topography for 400-grit burnished surfaces at different passes and locations. (**a**) A single pass 5 mm from the center; (**b**) a single pass 15 mm from the center; (**c**) four passes 5 mm from the center; (**d**) four passes 15 mm from the center.

Figure 6 shows the top surface microscopy for brass workpieces initially ground with 400 grit and then burnished for one pass (a) 5 and (b) 15 mm from the center line of burnishing. Figure 6a shows that ground marks almost disappeared on the burnished surface and the effect of burnishing starts to appear on the surface, particularly 5 mm around the burnishing center after one pass. In contrast, the base material starts to appear at the surface and grain boundaries are observed on the surface 15 mm from the burnishing center, as shown in Figure 6b. After the fourth pass, a totally new surface layer is formed (Figure 6c,d), where the ground marks observed after the first pass are completely removed. New scratches appeared on the new layer, which might be due to the low initial pin velocities at the new layer before the formation of the magnetic brush. As mentioned earlier, after the third and fourth passes, an increase in sample temperature was observed along with the presence of brass chips in a powder form on the surface of the sample, which helped obtain a new layer, as shown in Figure 6c,d.

### 3.3. Microroughness

**Figure 7:**
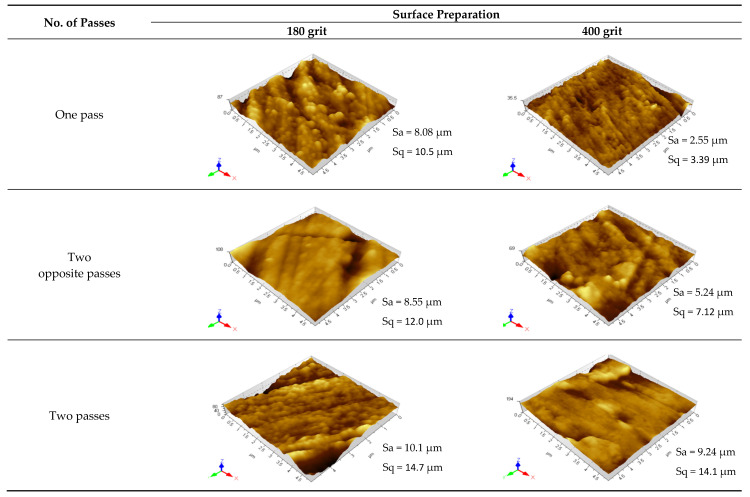

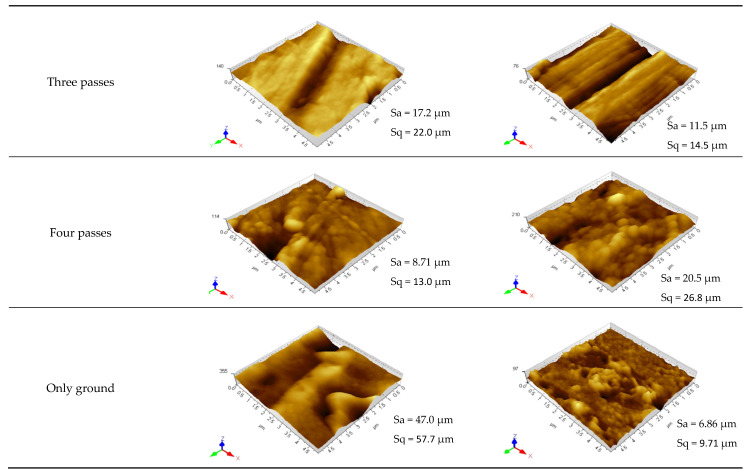
AFM topography images and roughness (Sa and Sq) for all sample.

**Figure 8:**
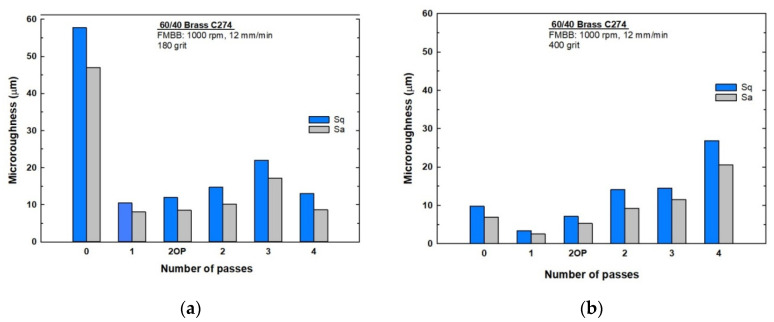
Microroughness results (Sa and Sq) for samples ground with (**a**) 180 grit and (**b**) 400 grit.

The parameters S_q_ and S_a_ were evaluated for all tested samples using the AFM results shown in Figure 7. S_q_, which is the standard deviation of the height distribution, and S_a_, which represents the arithmetic mean of the absolute values of profile heights, are the most widely used parameters in roughness measurements. All topography images were obtained in a 5 × 5 µm surface area using both contact and tapping modes. The visual differences in the topography surfaces of the samples are evident; however, a quantitative way to distinguish between these samples can be obtained from S_a_ and S_q_ data, as shown in Figure 8. The samples initially ground with 180 grit (Figure 8a) exhibited a significantly better surface microroughness reduction than the unburnished sample. The one-pass sample exhibited microroughness improvement over the ground sample by 82–83%. In the case of the two opposite passes, the improvement was 79–82%. These two cases exhibited the best two microroughness reductions among all the other passes for samples initially ground with 180 grit; this agrees with the microhardness improvement results. The microroughness improvements of the two-, three-, and four-pass samples are 75–79%, 62–63%, and 78–82%, respectively.

For samples initially ground with 400 grit (Figure 8b), only the samples corresponding to one pass and two opposite passes exhibited a better surface microroughness reduction compared with the unburnished sample. All other samples had higher microroughness than the unburnished sample, indicating that another process besides burnishing is involved in these samples. Generally, as the number of passes increases, the microroughness values increase, up to 176–199% in the fourth pass. The one-pass sample shows a microroughness improvement of 63–65%, whereas that of the two opposite passes is 24–27%. The two- and three-pass samples have higher microroughness values than that of the initial ground sample by 35–45% and 49–68%, respectively.

## 4. Discussion

### 4.1. Influence of Initial Surface Condition

The surface integrity of materials is an important requirement in the manufacturing industry because of its direct impact on properties such as fatigue and wear resistance [24]. It is strongly affected by several parameters such as plastic deformation, residual stresses, roughness, and cracks [25,26]. Thus, the final surface quality of any produced material is strongly influenced by the prior processing steps [27,28]. Correspondingly, the initial surface processing plays a major role in the final surface microstructure and in turn on the surface integrity [27]. The burnishing parameters used in the present study were selected based on the optimum surface quality, hardness, roughness, and corrosion resistance of a previous study conducted only on 180 grit of C274 brass samples [20], wherein the optimum parameters, which in turn correspond to the optimum force, were found to be 1000 rpm and 12 mm min^−1^. In this study, the decrease in microhardness and the increase in roughness of the samples initially ground with 180 grit and subjected to more than one pass, Figure 3a and Figure 8a, established that the optimum force obtained from the previous study is valid and that additional passes harm the surface quality. Several previous studies proved that increasing the number of burnishing passes using the optimum force leads to reduction in the hardness of the material owing to excessive work hardening [29].

Increased hardness due to burnishing is believed to result from the greater friction of the pin edges of the burnishing brush and the peaks of the workpiece surface texture. Peaks tend to deform easily where the gap between the peaks and valleys is sufficiently large to allow stretching. Owing to the complexity of the crystal structure in metals, as more slipping occurs owing to mechanical deformation, more obstacles tend to occur because various dislocation lines cross each other. This restriction of the dislocation motion makes the metal harder and stronger [30].

Hassan and Maqableh [31] proved that different initial surface hardness does not significantly affect the surface hardness after burnishing, as the amount of deformation is the same when the burnishing force is constant. In the present study, there was almost no difference between the microhardness values and uniformity between the prepared samples that were ground with 180 and 400 grit, as shown in Figure 3a,b. In contrast, the initial roughness shows a significant difference between ground samples with 180 and 400 grit on the final hardness and roughness for both S_q_ and S_a_, as shown in Figure 8a,b. The rougher surfaces of the 180-grit ground samples exhibit better hardness improvements than the rougher surfaces of samples ground with 400 grit for the different multi-pass FMMB. This is because of the higher amount of deformation and optimum force attained by the samples ground with 180 grit. When the optimum force is reached from the first pass, further passes do not increase the burnished sample’s hardness [29], and any additional passes reduce the hardness and impair surface quality because of excessive work hardening [32]. With a smoother surface using the same burnishing parameters, the improved hardness owing to burnishing was limited due to the lower deformation amount. This occurred because the gaps between peaks and valleys were small and the force used was lower than the optimum force needed for samples ground with 400 grit, and more passes were required to obtain further deformation and, thus, better hardness. Therefore, if the applied force is less than the optimum, more passes are recommended to have higher hardness [32]. Comparing the 400-grit samples to the 180-grit ones, Figure 3a,b, the maximum hardness values were obtained after the fourth pass, even though the values were 11% lower than those obtained in the first pass for 180-grit samples.

Rougher prepared surfaces are subjected to more mechanical deformation because of their higher peaks, and the FMMB process succeeds in flattening these peaks more than in smoother surfaces. In fact, the burnishing forces in FMMB are because of the constant strength of the permanent magnet and working conditions such as feed, rotational speed, and burnishing gab (angle of the pins). As aforementioned, these parameters were optimized based on the study by Alaskari et al. [20], wherein the brass surface was initially ground only with 180 grit and the optimum burnishing force was reached using these parameters. However, these parameters were kept constant throughout the burnishing process for samples ground with 400 grit. Thus, for these samples, these parameters do not result in the optimum force required to perform the required burnishing process and to obtain better roughness. By increasing the number of passes, more plastic deformation of the surface took place, which increased the work hardening of the earlier deformed surface. Subsequently, the surface tended to resist further deformation and flaking occurred instead [33], harming the previously achieved surface roughness.

### 4.2. Influence of the Number of Burnishing Passes

**Figure 9:**
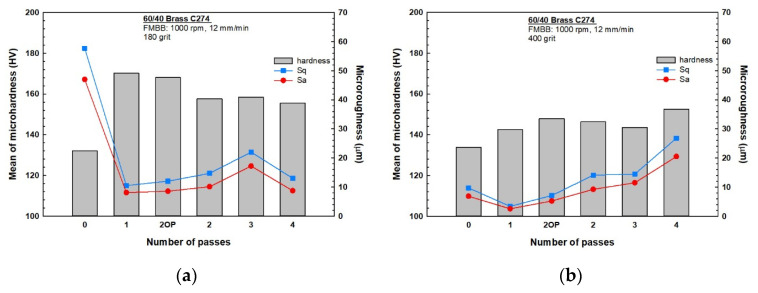
Microhardness and microroughness of samples ground initially with (**a**) 180 grit and (**b**) 400 grit.

The factors such as surface condition, roughness, microhardness, and process uniformity should be studied collectively to clearly understand the present multi-pass FMMB process. For initially ground samples with 180 grit, Figure 9a shows that all single- and multi-pass samples exhibit better hardness and roughness but with lower process uniformity, as shown in Figure 3a, than the ground samples. As the number of passes increases, the samples become less hard and the surface becomes rougher in comparison with the previous pass. When the optimum force is reached from the first pass, with farther passes the hardness values are reduced owing to excessive work hardening [29]. In addition, the pin shape, which has a small contact surface area, facilitates the flaking process, especially after reaching the optimum force.

After the first pass of the sample initially ground with 180 grit, the improvement of hardness and roughness was clearly observed along with some non-uniform process. Scratches and, in turn, non-uniformity were present nearer to the center of the burnishing process, as shown in Figure 5a, where burnishing effects appear stronger and more uniform away from the center, as shown in Figure 5b. These scratches are attributable to the low initial pin velocities and non-uniformity at the location near the burnishing center before the formation of the magnetic brush. The two opposite-pass samples showed almost no reduction in microhardness and increased roughness due to severe work hardening. However, they showed much better process uniformity. After reaching optimum values, any additional force decreased the hardness because of surface flaking, as explained by Babu et al. [34]. The repeating plastic deformation process, owing to multiple passes on the surface layer, increases the work hardening of the earlier deformed surface; in turn, this initiate flaking, leading to higher surface roughness as explained by El-Taweel and El-Axir [33]. The two-pass sample showed even further impaired microhardness, roughness, and process uniformity, indicating a greater amount of surface flaking toke. The three-pass sample exhibited a small improvement in microhardness and process uniformity but at the expense of a large increase in roughness (70%). This indicated both minor burnishing processes for the new surface layer and high surface flaking for the previously severely deformed surface. The four-pass sample greatly improved the roughness (by 49%) with almost no change in microhardness and uniformity for the new surface. This indicates more of a burnishing process after flaking during the previous pass, with only minor subsequent surface flaking.

For the initially prepared samples that were ground with 400 grit, all single- and multi-pass samples exhibited better microhardness than the ground sample, as shown in Figure 9b, but only one pass and two opposite passes produced better roughness and process uniformity than the ground sample, as shown in Figure 3b. In addition, the microhardness and roughness increase with the number of passes, which contradicts the mechanism of the burnishing process, in which the microhardness and roughness are inversely proportional. The increase in hardness indicates that the applied force is less than the optimum required to perform the burnishing, and further passes are needed to reach the maximum force, as explained by Loh and Tam [35]. In addition, higher process non-uniformity was noted as the number of passes increased owing to over-surface hardening at some sample locations. This leads to surface flaking, consequently increasing the coefficient of friction [36] and thus increasing roughness. This action can also be explained by over-hardening and the consequent flaking of the surface layers due to the repeated passage of burnishing brushes over certain surface areas, which was found to be in good agreement with the findings of Revankar et al. [37]. After one pass of burnishing, all surface properties were enhanced and grain boundaries started to appear on the surface of the sample 15 mm away from the burnishing center, as shown in Figure 6b. The improvement in hardness, roughness, and uniformity indicates that a uniform burnishing process occurred. For the two-opposite-pass sample, a slight increase in microhardness with better process uniformity and increased roughness indicates that the burnishing process and surface flaking took place for some parts of the sample surface. For the two-pass sample, a small increase in microhardness with less uniformity and a major increase in roughness compared to the previous pass indicates a non-uniform high surface flaking mechanism and that some burnishing took place. The three-pass sample exhibited almost no change in microhardness and process uniformity, in contrast to the previous pass; however, higher roughness indicates a further surface flaking mechanism with almost no burnishing. For the four-pass sample, an increase in microhardness with lower uniformity and a large increase in roughness occurred, indicating non-uniform burnishing and surface flaking mechanisms. Figure 6c,d depict the appearance of a new surface after the fourth pass, indicating total removal of the previous pass layer by flaking after repeated plastic deformation, leading to increased work hardening of the deformed surface.

### 4.3. Influence of Strain Reversal

The Bauschinger effect (BE) [38] holds that, when plastic deformation takes place in a specific direction within a metal, the flowing stress should be lower for consequent loading in the opposite direction than in the same direction. Therefore, the BE is applicable for parts with respect to the deformation path and history, depending on the hardening behavior. The BE appears when dislocation pileups at microstructural grain boundaries and second-phase particles generate distant stresses that can contribute to deformation through reverse loading [39,40]. Therefore, the material characteristics and properties depend on the applied forces and direction between the first and second strains [41]. In the current study, it was found that using burnishing with two opposite passes, based on strain reversal, allows deformation of the surface layer again and, to a greater extent, with less degradation (flaking) in comparison with using same-direction passes. This explains why two opposite passes are preferable rather than two same-direction passes, where the surface roughness, process uniformity, and hardness showed almost no change or even improved compared to the single-pass case. The two opposite passes always show greater improvement of microhardness and roughness than two same-direction passes, where these improvements are greater for 180-grit samples than for 400-grit samples, as shown in Figure 9a,b. In addition, the two-opposite-pass treatment always produces far better uniformity than that of two same-direction passes, and even better than the single pass where process uniformity with the 400-grit prepared samples is much better than that of the 180-grit prepared samples, as shown in Figure 3a,b. In terms of the objective of our study, one can argue that it is not enough to have a high hardness value, but rather that this value should be accompanied by a uniform distribution along the burnishing surface.

## 5. Conclusions

In this study, the FMBB process was modified to create a multi-pass FMBB process in order to improve the surface properties and uniformity of 60/40 brass. The experimental results demonstrated the effects of several different factors on surface characteristics. The conclusions drawn are as follows:

- The initial surface roughness plays a major role in the final surface in terms of surface integrity; as the applied force is optimal, larger deformation occurs on a rougher surface.
- Generally, increasing the number of passes increases the surface roughness owing to surface deformation resistance and over-hardening, which in turn changes the process to initiate flaking.
- The rougher-surface brass samples provide better final hardness improvements, and as the number of passes increases, the samples become less hard and rougher but with better uniformity in comparison with the previous pass owing to over-hardening and flaking mechanisms.
- The smoother surface of the brass samples delivers a limited improvement in hardness, because the exerted force is less than the optimum burnishing force.
- As the number of passes increases for smoother-surface samples, the microhardness, surface roughness, and non-uniformity increase owing to over-surface hardening because of repeated burnishing at some sample locations. This leads to surface flaking and an increase in the coefficient of friction.
- Opposite burnishing passes are found to be beneficial to the machined surface owing to the reverse strain mechanism, which has shown better surface roughness, process uniformity, and microhardness improvements than using two same-direction passes.
- Two opposite passes are preferable, rather than one pass wherein the surface roughness and microhardness showed almost no change with high process uniformity.

## Figures and Tables

**Table 1:** Chemical compositions (wt. %) of brass workpieces and stainless steel pins.

Element	C274 (brass)	SS303 (pins)
Cu	60.70	0.55
Zn	39.20	–
Fe	–	70.81
Cr	–	18.15
Ni	0.13	8.14
Mn	–	1.60
Mo	–	0.28

**Table 2:** Sample procedure for all multi-pass burnishing processes.

Initial Grinding Grit	Sample Name	Process			
180	One Pass	First Pass			
	Two opposite passes	First pass	Second pass opposite direction		
	Two passes	First pass	Second pass		
	Three passes	First pass	Second pass	Third pass	
	Four passes	First pass	Second pass	Third pass	Fourth pass
400	One pass	First pass			
	Two opposite passes	First pass	Second pass opposite direction		
	Two passes	First pass	Second pass		
	Three passes	First pass	Second pass	Third pass	
	Four passes	First pass	Second pass	Third pass	Fourth pass
